# Firearm use risk factors and access restriction among suicide decedents age 75 and older who disclosed their suicidal intent

**DOI:** 10.3389/fpubh.2023.1255519

**Published:** 2023-11-03

**Authors:** Namkee G. Choi, C. Nathan Marti, Bryan Y. Choi

**Affiliations:** ^1^Steve Hicks School of Social Work, University of Texas at Austin, Austin, TX, United States; ^2^Department of Emergency Medicine, Philadelphia College of Osteopathic Medicine and BayHealth, Dover, DE, United States

**Keywords:** suicide intent disclosure, firearm suicide, suicide methods, means restriction, suicide intent disclosure, suicide prevention

## Abstract

**Background and aims:**

A majority of older adult suicide decedents used firearms. In this study, we focused on suicide decedents age 75+ who disclosed their suicidal intent within a month of their injury/death to examine demographic and clinical characteristics associated with firearm use and firearm access restriction attempts by their family members.

**Methods:**

The 2017–2019 U.S. National Violent Death Reporting System provided data (*N* = 1,734 suicidal intent disclosers; 1,476 males and 258 females; 21.4% of decedents age 75+). Generalized linear model (GLM) for a Poisson distribution with a log link was used to examine firearm use risk factors. Firearm access restriction attempts by decedents' family members were examined based on coroner/medical examiner and law enforcement (CME/LE) reports.

**Results:**

Nearly three quarters of disclosers disclosed their intent to family members, and 82.9% of males and 27.5% of females used firearms. GLM results showed males, non-Hispanic white people, and residents in the South and West regions had higher likelihood of firearm use. CME/LE reports of 140 out of 1,294 firearm decedents included narratives related to firearm restriction attempts or lack thereof. Firearm access restrictions were not attempted in 65 cases because family members did not take suicidal intent disclosure seriously or because decedents promised no self-harm. Partial or complete removal of firearms in 75 cases were not effective as decedents had hidden a firearm or purchased a new one. Others used different lethal methods.

**Implications:**

The findings indicate a need for: (a) training family members of older adults who are at risk of suicide in effective means safety/access restriction and strategies to prevent means substitution; (b) more comprehensive legislative reforms reducing access to firearms by those at risk of suicide; and (c) more comfort and palliative care and counseling for psychosocial risk factors.

## Introduction

In 2021 alone, 48,183 people in the United States (US) died by suicide, which was one death every 11 minutes, and 26,328 of them were firearm suicides (1, 2). Continuing the trend over the past two decades, males age 75 and older had the highest suicide rate (42.2 per 100,000 population in 2021) of all age groups (3). High suicide rates among older adults have been attributed to the fact that they engage in suicidal behaviors with greater premeditation, lethality of intent, and lethality of method, firearms in particular, than do younger adults (4, 5).

While firearms are the most and second most frequently used suicide method among all adult males and females, respectively, in the US, older adults are far more likely to use firearms than younger adults (6–9). A study of suicide decedents in the 2017–2019 National Violent Death Reporting System (NVDRS) showed that firearms were used in 55 and 30% of all adult male and female suicides, respectively, but they were used in 75 and 34% of male and female suicides, respectively, in the 65+ age group (6). Among older male suicide decedents, 70.2, 88.8, and 80.3% of those age 65–74, 75–84, and 85+, respectively, used firearms (6). Firearm suicides among those age 65+, disproportionately affecting non-Hispanic white males, increased by 49% between 2010 and 2018, from 4,276 in 2010 to 6,375 in 2018, and continued to increase further in 2020 and 2021 (5, 10, 11), showing that 18 older adults die by firearm suicide every day.

The high rate of firearm suicides compels firearm-related means safety and access restriction as essential strategies for suicide prevention. While these strategies in the US mostly involve firearm safety counseling training of healthcare professionals who interact with those at risk of suicide (12–20), the effects of their trainings on preventing suicide at an individual level have not been examined. However, a study of state-level data found that household firearm ownership and percent with loaded guns and ready access to guns were significantly positively associated with the rates of firearm suicide and suicide by all methods, whereas strictness of gun regulation reduced suicide rates (21). Given that 42% of those age 65+ personally own a gun or live in a household with a gun (22), gun safety for all and access restriction for older adults with suicidal thoughts and intent are indeed necessary for saving lives.

Research has shown that a little over one fifth of older suicide decedents age 65+ disclosed their suicidal intent within a month of their death, and most of them disclosed their intent to spouse/partner, other family members, or friends/neighbors (23, 24). This rate of disclosure appears to be lower than the rates (40.8–44.5%) found in meta-analyses of studies of all age groups of adult suicide decedents (25, 26). Although the difference may be in part due to the different time frames (e.g., within a month vs. within a year) used in different studies, the lower disclosure rate among older adults may be due to fatalistic views that nothing can be done, social isolation (i.e., no one to disclose to), concerns about unwanted hospitalization or other interventions especially among those with a high degree of wish to die, or not wanting to overburden family (27–30). Some may also engage in “dissembling,” keeping everything inside or keeping the façade (31, 32). Older adults may also disclose their suicidal intent out of a sense of duty to inform their family and as a way to prepare their family members for their death (i.e., not as a surprise), which may be part of their suicide plan.

Studies have also shown that another reason for intent disclosure, to mostly one's confidants and others in the informal social support network, was help-seeking (33–35). For older adults who suffer from terminal illness, chronic pain, and/or functional disability, and/or mental health problems, disclosing their suicidal intent to their informal support systems and/or healthcare providers may also be a way of expressing their suffering and seeking help and support. Regardless of disclosure reasons, disclosure of suicidal intent provides a great opportunity to intervene and manage risks and to reduce premature mortality from suicides. Since most older adults' disclosures are made to their family and other informal support systems, these informal systems, especially family members, have an important role in managing older adults' access to suicide means, especially firearms. However, little research has been done on their involvement in access restriction.

In this study, we focused on suicide decedents age 75+ who had disclosed their suicidal intent within a month of their injury/death to first examine demographic and clinical factors, including suicide precipitants, associated with firearm vs. other suicide method use. We then described firearm access restriction attempts, or lack thereof, by family members, as noted in the coroner/medical examiner and law enforcement agency reports. Our focus on those age 75+ was based on the fact that the suicide rates among males in this age group continued the increasing trend despite the brief dip in suicide rates among all other age groups in 2020 (36) and that this age group has the highest rates of using firearms as the suicide method (6). This study's findings will provide insights into firearm use among suicide decedents age 75 who disclosed their suicide intent and the role of family in restricting firearm access.

## Materials and methods

### Data source

Data came from the 2017–2019 NVDRS, which is the only state-based violent death reporting system in the US that provides information and context on when, where, and how violent deaths occur and who is affected (37). NVDRS links data from death certificates and reports from coroners/medical examiners (CME) and law enforcement (LE) agencies on cases of violent deaths–suicides, homicides, deaths from legal intervention (i.e., victim killed by LE acting in the line of duty), deaths of undetermined intent, and unintentional firearm deaths. CME/LE reports are from the injury/death scene, ongoing investigations, or family/friend accounts and often serve as the basis of the circumstances of death and the NVDRS variables that were “calculated” (coded “Yes” when endorsed by the CME and/or LE reports vs. “No/not available/unknown”).

We used 2017–2019 NVDRS data because the number of participating states increased from 27 in 2016 to 37 in 2017 and to 43 states, the District of Columbia, and Puerto Rico in 2019, although not all states provided complete data for all 3 years (38). Our preliminary analysis showed that some important results vary depending on the number of participating states. We were granted access to de-identified NVDRS data for this study by the Centers for Disease Control's NVDRS-Restricted Access Data (RAD) review committee.

The 2017–2019 NVDRS provided data on a total of 94,457 (74,042 male, 20,412 female, and 3 unknown sex) suicide decedents, ages 18–105 at the time of death. Of these, 8,120 were age 75+, and 21.4% or 1,734 (1,476 male and 258 female) decedents disclosed their suicidal intent. These 1,734 decedents became this study's focus. This study based on de-identified data on deceased individuals was exempt from the authors' institutional review board's review.

### Measures

#### Suicidal intent disclosure

In NVDRS, suicidal intent disclosure was defined as (1) explicit (e.g., “I plan to go to my cabin with my gun and never come back”) or indirect (e.g., “I know how to put a permanent end to this pain”) communication of suicidal intent to another person via verbal, written, or electronic communications within a month (or recently) before suicide, or (2) a separate suicide attempt within a month of the suicide. It excludes any disclosure only at the moment of the suicide (i.e., when there was no opportunity to intervene to stop the suicide). Non-disclosure was defined as an absence of disclosure or unknown disclosure status.

#### Suicide methods

These were identified from the International Classification of Diseases, 10^th^ Revision (ICD-10), codes for intentional self-harm (X60–X84) for underlying cause of death in death certificates and/or from the underlying cause descriptions in CME reports. They included the following: firearms; hanging/suffocation; poisoning due to any type of alcohol/drug/medicine/chemical overdose or with gas (e.g., carbon monoxide, nitrogen); laceration/sharp instruments; blunt objects; jumping from heights; contact with moving objects (train/other vehicles); drowning; and other (fire, hypothermia, electrocution, starvation, dehydration, not adhering to or refusing medical care, other specified but not elsewhere classified methods, and unspecified methods). We classified them into four categories in this study: firearms, hanging/suffocation, poisoning, and all other methods.

#### Demographic variables

Data on age at the time of death (75–84 and 85+), sex, race/ethnicity, level of education, marital status, military service history, US Census region of residence, and injury location (descriptive purpose only in this study) were obtained from the death certificates and CME/LE reports.

#### History of suicide attempts

This referred to any previous suicide attempt before the fatal incident (i.e., including any in the past month), regardless of the severity and injury status.

#### Mental health and substance use problems/addiction

Without the need for any indication that they directly contributed to the death. Mental health problems included: (a) depressed mood at the time of death (without the need for a clinical diagnosis), and (b) any diagnosed mental illness [disorders and syndromes listed in DSM-5 (39)] at the time of death. Substance misuse/addiction problems included: (a) alcohol problems, and (b) other substance misuse (e.g., prescription drug misuse, chronic/abusive/problematic marijuana use, any use of other illicit drugs or inhalants).

#### Mental health/substance use treatment receipt at the time of injury

This was coded “Yes” if the decedent was in treatment (e.g., had a current prescription for a psychiatric medication, saw a mental health professional within the past 2 months, or participated in treatment for substance use such as outpatient treatment or self-help group) at the time of the injury.

#### Suicide precipitants

These were based on CME/LE reports and included the following: (a) physical health problems (coded “Yes” only if any diagnosed or perceived physical health problem [e.g., terminal disease, debilitating condition, chronic pain] was relevant to the death [e.g., “despondent over recent diagnosis of cancer” or “complained that he could not live with the pain associated with a condition” even if the condition may not have been diagnosed or existed]); (b) recent suicides or other deaths of spouse/other family/friends or a traumatic anniversary; (c) relationship problems (conflict with an intimate partner and/or other family members, arguments, other family stressors, caregiver burden, or abuse by a caregiver); (d) job/finance/housing problems; and (e) criminal/civil legal problems.

### Analysis

All statistical analyses were performed using Stata/MP 18. First, we used χ^2^ and Fisher's exact tests to compare demographic and clinical characteristics between those who used firearms and those who used other methods. Second, we fit a generalized linear model (GLM) for a Poisson distribution with a log link to examine the associations between demographic/clinical factors and firearm vs. other method in multivariable analysis. We fit a GLM rather than a logistic regression model because odds ratios exaggerate the true relative risk to some degree when the event (i.e., firearm use in this study) is a common (i.e., >10%) occurrence (40). As a preliminary diagnostic, we used variance inflation factor (VIF), using a cut-off of 2.50 (41), from linear regression models to assess multicollinearity among covariates. VIF diagnostics indicated that multicollinearity was not a concern. GLM results are reported as incidence rate ratios (IRRs) with 95% confidence intervals (CIs). Significance was set at *p* < 0.05.

Third, firearm safety and access restriction attempts by family members, as described in the CME/LE reports (mostly 100–350 words each), were examined using the following four steps for identifying, retrieving, and analyzing relevant content: (a) We reviewed all available CME/LE narratives for those who died by firearms following disclosure of their suicidal intent to gain preliminary understanding of the types of descriptions related to firearm access restriction or lack thereof; (b) Based on our review, we compiled a comprehensive list of the terms (words or phrases) that were contained in these descriptions; (c) The word search function in SPSS v.28 was used to identify cases containing these terms; and (d) All CME/LE narratives of the identified cases were reviewed again to better understand the injury/death circumstances and generate themes related to firearm safety and restriction. Because direct quotes from CME/LE reports are not allowed in NVDRS, we compiled similar and dissimilar circumstances and developed composite summary descriptions and did not provide any other specific characteristics when reporting circumstances pertaining to a single decedent.

## Results

### Characteristics of suicide decedents age 75+ who disclosed suicidal intent by suicide methods

Table 1 shows that nearly three quarters of all intent disclosers disclosed their suicidal intent to their spouse/partner or other family members, one tenth to friends/neighbors, about 7% to a healthcare provider, and the rest to others. Of the disclosers, 74.6% used firearms and 25.5% other methods. Of the latter, nearly one half used poisoning and nearly one third used hanging/suffocation. Overall, 85.1% of all discloser-decedents were male; however, 94.5% of firearm users, compared to 57.0% of other method users, were male. Additional analysis found significant sex difference in firearm users (82.9% of males and 27.5% of females; Pearson χ^2^ = 355.18, df = 1, *p* < 0.001; refer to Supplementary Table 1 showing sex differences in the study variables in all disclosers and then in firearm users).

**Table 1:** Characteristics of suicide decedents age 75+ who disclosed suicidal intent within last month by suicide method.

**N (%)**	**All**	**Firearm**	**Other method**	**p**
	**1,734 (100%)**	**1,294 (74.6%)**	**440 (25.4%)**	
**Disclosed suicidal intent to whom (%)** ^a^				
Previous or current intimate partner and/or other family member	73.1	73.9	70.7	0.192
Friend or neighbor	10.9	11.5	9.1	0.184
Healthcare worker	7.7	6.1	12.5	< 0.001
Other	13.4	13.5	13.0	0.808
**Suicide method**				< 0.001
Firearms	74.6	100	0	
Hanging/suffocation	8.0	0	31.4	
Poisoning	12.4	0	48.8	
Other^b^	5.0	0	19.8	
**Sex**				< 0.001
Male	85.1	94.5	57.0	
Female	14.9	5.5	43.0	
**Age group (%)**				
75–84 years	67.9	67.9	67.7	0.938
85+ years	32.1	32.1	32.3	
**Race/ethnicity (%)**				< 0.001
Non-Hispanic white	93.0	95.9	84.6	
Black/African American	1.8	1.8	2.1	
Hispanic	2.4	1.4	5.2	
Asian/Pacific Islander	2.3	0.5	7.7	
Other	0.5	0.5	0.5	
**Education (%)**				< 0.001
= < High school	57.2	60.2	48.2	
Some college/associate's degree	17.0	16.8	17.3	
Bachelor's degree or higher	23.4	20.2	32.5	
Unknown	2.5	2.7	2.0	
**Marital status (%)**				< 0.001
Married	45.3	48.0	37.5	
Widowed	31.0	29.7	34.8	
Divorced/separated	19.0	18.4	20.9	
Never married/non-specified single	3.9	3.2	5.9	
Missing	0.7	0.7	0.9	
Military service history (%)	48.3	56.1	25.2	
**Census region (%)**				< 0.001
Northeast	12.2	10.0	18.9	
Midwest	25.8	27.0	22.5	
South	26.1	28.7	18.6	
West	35.3	34.3	38.2	
Puerto Rico	0.5	0.1	1.8	
**Injury location (%)**				0.003
At home	87.1	88.6	83.0	
Not at home	12.9	11.4	17.0	
**History of suicidal thoughts and plans**	63.4	62.0	67.5	0.039
**History of suicide attempt (%)**	10.4	6.0	23.4	< 0.001
**Depressed mood at the time of injury**	45.0	44.0	47.0	0.346
**Any diagnosed mental illness**^c^ **(%)**	36.0	31.5	49.5	< 0.001
Depressive disorder/dysthymia (%)	26.7	23.6	35.9	< 0.001
Bipolar disorder (%)	1.6	1.0	3.4	0.001
Anxiety disorder (%)	6.5	4.9	11.1	< 0.001
Post-traumatic stress disorder (%)	0.9	0.9	0.7	0.773
**Alcohol problem/addiction (%)**	5.1	5.4	4.3	0.453
**Other substance use problem**^d^ **(%)**	1.5	1.1	2.7	0.021
**Mental health/substance use treatment receipt at the time**	18.7	15.7	27.7	< 0.001
**of injury**^e^ **(%)**				
**Suicide precipitating factors (%)**				
Physical health problem^f^	69.7	73.5	58.6	< 0.001
Suicide/death of spouse/family or traumatic anniversary	15.2	14.6	16.8	0.281
Relationship problem^g^	12.5	12.5	12.5	1.000
Financial problem or eviction/loss of housing	6.2	6.0	6.8	0.494
Criminal or civil legal problem	2.1	2.2	1.6	0.561
**CME/LE narrative search results related to physical health problems**				
Pain	27.1	27.7	25.2	0.321
Cancer	23.1	25.3	16.4	< 0.001
Heart disease	16.5	17.2	14.3	0.159
Dementia	12.0	11.5	13.4	0.308
Chronic obstructive pulmonary disease	6.5	6.6	6.1	0.823
Parkinson's	3.0	2.9	3.4	0.522
Worry about becoming a burden	5.9	5.8	6.1	0.815
Refusal of nursing home placement	5.0	4.9	5.0	1.000
Low quality of life	1.5	1.3	2.0	0.264
Lived alone	2.4	2.6	2.0	0.720

*P*-values are calculated based on Pearson's χ^2^ tests or Fisher's exact tests and refer to any differences between firearm and other method users. ^a^Categories are not mutually exclusive. ^b^Jump from a high place, blunt force from moving vehicle/train/other, sharp or blunt object, drowning, smoke/fire/flame/electrocution/hypothermia, other means, or unknown. ^c^Including those disorders and syndromes listed in the Diagnostic and Statistical Manual of Mental Disorders, Fifth Edition (DSM-5) with the exception of alcohol and other substance dependence. ^d^Including illicit drug use, even if addiction or abuse is not specifically mentioned. The exception to this is marijuana use. For marijuana, the use must be noted as chronic, abusive, or problematic. ^e^Inclusive of pharmacotherapy, psychotherapy/counseling, any class (e.g., anger management) attendance, any facility-based care, and alcohol or narcotics anonymous. ^f^Including any terminal/other illness, debilitating condition, chronic/acute pain, or other physical/functional issue (perceived, or diagnosed) that were relevant to suicide. ^g^Problems with intimate partner and/or other family/relatives, other family stressors, caregiver burden, arguments, or abuse by a caregiver.

Table 1 also shows that firearm users, compared to other method users, included significantly higher proportions of non-Hispanic white people, those with less than college education, those who were married, those with a military service history, and residents in the South region. On the other hand, firearm users had lower proportions of those with previous histories of suicidal thoughts, plans, or attempt, any mental disorders, and substance use problems, and those who were receiving mental health/substance use treatment at the time of injury. Only 18.7% of all disclosers were receiving any mental health/substance use treatment (mostly medications) at the time of injury.

Of suicide precipitants, 73.5% of firearm users and 58.6% of other method users had physical health problems (Pearson χ^2^ = 33.34, df = 1, *p* < 0.001). Additional analysis, using the word search function of SPSS v.28, of the CME/LE narratives related to physical health problems identified “pain” (e.g., not being able to relieve chronic pain despite all treatments and its effect on sleep and functioning) in 27.7% of firearm users and 25.2% of other method users; cancer in 25.3% of firearm users and 16.4% of other method users; any heart disease in 17.2% of firearm users and 14.3% of other method users; and dementia (e.g., being upset following a dementia diagnosis and/or about worsening symptoms) in 11.5% of firearm users and 13.4% of other method users. Except cancer, differences in these and other health conditions and related circumstances did not significantly differ between firearm users and other method users. The two groups did not significantly differ on other suicide precipitants.

### Associations between firearm use and demographic and clinical factors: Multivariable findings

Table 2 shows that firearm use, as opposed to other method use, was significantly positively associated with male sex (IRR = 2.58, 95% CI = 2.00–3.32), non-Hispanic white race/ethnicity (IRR = 1.43, 95% CI = 1.08–1.89), and residence in the South (IRR = 1.31, 95% CI = 1.07–1.61) compared to the Northeast region. Residence in the West was also marginally significant. Firearm use was negatively associated with a college education (IRR = 0.87, 95% CI = 0.76–0.99) and a previous suicide attempt history (IRR = 0.70, 95% CI = 0.55–0.89). All other factors, including mental health and substance use problems and treatment receipt, suicide precipitants, and to whom the intent was disclosed, were not significant.

**Table 2:** Associations of firearm use with demographic and clinical characteristics of suicide decedents age 75+ who disclosed suicidal intent within last month: Generalized linear modeling results.

**Model parameter**	**Firearm use vs. other method use IRR (95% CI)**
**85**+ **age group vs. 75–84 age group**	0.98 (0.86–1.11)
**Male vs. female**	2.58 (2.00–3.32)^***^
**Non-Hispanic white vs. all other race/ethnicity**	1.43 (1.08–1.89)^*^
> **Bachelor's degree vs. less education**	0.87 (0.76–0.99)^*^
**Marital status: vs. Married/cohabiting**	
Widowed	0.97 (0.83–1.14)
Divorced/separated/never married/other	0.99 (0.86–1.14)
Military service history vs. no military service	1.10 (0.98–1.24)
**Census region: vs. Northeast**	
Midwest	1.17 (0.96–1.44)
South	1.31 (1.07–1.61)^**^
West	1.21 (0.99–1.48)^‡^
**History of suicide attempt vs. no history**	0.70 (0.55–0.89)^**^
**Depressed mood vs. none depressed mood at the time of injury**	0.96 (0.86–1.08)
**Any diagnosed mental illness vs. none**	0.94 (0.81–1.10)
**Alcohol other substance use problem/addiction vs. none**	0.96 (0.75–1.21)
**Mental health/substance use treatment receipt at the time of**	0.94 (0.78–1.14)
**injury vs. none** ^a^	
**Suicide precipitating factors (%)**	
Physical health problem^b^	1.10 (0.96–1.25)
Suicide/death of spouse/family or traumatic anniversary	1.00 (0.84–1.19)
Relationship problem^c^	1.03 (0.86–1.24)
Financial problem or eviction/loss of housing	1.01 (0.79–1.17)
Criminal or civil legal problem	1.09 (0.75–1.60)
**To whom disclosed suicidal intent**	
Intimate partner and/or other family	1.00 (0.78–1.28)
Friend or neighbor	1.04 (0.79–1.36)
Healthcare worker	0.83 (0.63–1.09)
Other	0.97 (0.76–1.24)
N = 1,725	

Nine cases from Puerto Rico were excluded. ^*^p < 0.05; ^**^p < 0.01; ^***^p < 0.001; ^‡^*p* = 0.058.

### CME/LE narratives related to firearm access restrictions following suicidal intent disclosure

Of 1,294 decedents who used firearms, 48 had neither CME nor LE report. Of the CME/LE narratives available for 1,246 decedents, only those pertaining to 140 decedents (11% of 1,246 firearm decedents) included any descriptions related to firearm safety measures or access restriction attempts by family members. CME/LE narratives did not show any other informal support system's (e.g., friend or neighbor) involvement in access restriction attempts.

Table 3 illustrates 11 themes that were generated from the 140 case narratives and the circumstances for each theme: (a) no access restriction attempt; (b) not enough time to remove firearms; (c) lack of awareness of decedents' possession of firearms; (d) no means restriction attempt given the presumed inevitability of suicide; (e) no family or living with a disabled spouse; (f) decedents' refusal to give up firearms; (g) initial removal but later returns; (h) inability to find firearms; (i) partial removal; (j) purchase of a new firearm; and (k) means substitution. In sum, firearm access restriction was not attempted for various reasons in 61 out of 140 cases, and partial or complete removal of firearms in 71 cases did not have the intended effect. The remaining 8 cases were those of means substitution; four cases used firearms when other means were taken away, and four others used other means when firearms were removed.

**Table 3:** Contents related to non/restrictions to firearm access following suicidal intent disclosure in CME/LE reports.

**Themes**	**Number (male; female)**	**Circumstances**
No access restriction as it was considered unnecessary	(26, 0)	• Family did not take the decedent's suicidal intent disclosure seriously because similar statements in the past were not acted upon. • Family was not worried as suicidal intent was only vaguely expressed. • While decedents had expressed their suicidal intent, they promised never to hurt themselves or stated that it was a joke. • Family never thought that the decedent would actually shoot himself (for insurance-related reasons and family's sake). • EMS or a doctor advised family to remove firearms, but they were not removed when the decedents promised no self-harm.
Not enough time to remove firearms	(16, 0)	• There was not enough time to attempt restrictive action as the disclosure was made so close to the time of injury. Some families lived hours away or out of state. • Family was planning on removing the firearms, but the decedents injured themselves before the removal.
Not aware of decedents' firearm possession	(4, 1)	• Family was unaware that the decedents owned any firearm. • Family thought that the decedent did not own a gun and would not be able to go to a gun store due to his inability to drive. • Decedent had told family that he only had a toy gun.
No removal given inevitability	(3, 0)	• Family members knew that suicide would be inevitable and did not attempt to remove firearms (The decedent carried a firearm everywhere he went). • Decedents discussed suicide with family and had affairs in order after a doctor denied their requests for assisted suicide.
No family or living with a disabled spouse	(3, 0)	• No one to remove as the decedent lived alone and had no family who could check on him. • Decedent's spouse was too sick/disabled to remove firearms.
Decedents' refusal to give up firearms	(3, 0)	• Decedents refused to give up firearms, insisting that they were for home protection or shooting critters.
Initially removed but returned firearms	(3, 0)	• Decedent asked spouse to get out a gun so that he could give it to someone else as a gift, but used it when he had the gun. • When family removed guns, the decedent became angry and the family had to return it to deescalate the situation.
No removal due to inability to find them	(2, 0)	• Family was aware that the decedent owned a firearm and tried to remove it, but could not find where the decedent kept it.
Partial removal	(45, 4)	• Family thought that they had removed all firearms (sometimes with the decedents' consent), but the decedent apparently kept one hidden. • Family removed one firearm, but could not find the second one. • Family moved a firearm from its original storage, but the decedent found it. • Removed handguns, but not rifles. • Family removed firearms that were easily accessible but left ones that were stored in a place (e.g., basement, high up in a wall cabinet) where they thought the decedents would not be able to reach due to mobility issues or other physical limitations. Decedents somehow accessed them. • Family placed firearms in a locked storage box or a pad-locked room; decedents used a hammer to pry open the box or a padlock. • Spouse removed all ammunition and did not know how the decedent was able to obtain it. • No gun at home, but the decedent used one at his business. • Spouse gave a firearm to a law enforcement agent when the decedent made statement of self-harm due to progressive dementia, and did not know where/how the decedent got another. • Law enforcement agent took firearms away 4 months before when the decedent threatened suicide; the decedent had another one.
Decedents purchased firearms close to injury time	(20, 2)	• Family removed all firearms, but decedents purchased new firearms. (Some families did not think that the decedent would be able to go to a gun store, but they found new purchase receipts following the injury.) • Some decedents had not owned a firearm, but receipts showed that they purchased one close to the injury time (e.g., 4 h or a day prior).
Means substitution	(8, 0)	• Four decedents died by hanging or by carbon monoxide poisoning after their family removed guns. • Four decedents died by firearms after family removed other means (knives, sleeping pills, other medications) that the decedents were trying to use or used in their previous suicide attempt.

*No attempt at access restriction* (26 male decedents): In these cases, family members did not take the decedents' disclosure of suicidal intent seriously since the decedents had a history of making similar statements or the decedents assured that they would not act upon their intent. Some families did not follow healthcare professionals' advice to remove firearms when the decedents promised no self-harm.

*Not enough time to remove firearms* (16 male decedents): These decedents injured themselves before their families had a chance to remove firearms. Some families lived hours away or in different states.

*No knowledge of firearm possession* (4 male and 1 female decedents): These decedents' families were unaware that the decedents owned any firearms or thought that they would not be able to go to a gun store due to lack of transportation.

*No removal due to inevitability* (3 male decedents): These decedents' families did not attempt to remove firearms as they knew that suicide would be inevitable and/or respected the decedents' choice. In the case of two decedents, families decided not to stop their suicide after doctors denied the decedents' request for assisted suicide.

*No one to remove firearms* (3 male decedents): One decedent had no family and the other's spouse was too sick and disabled to remove firearms from the decedent. CME/LE reports specifically mentioned “lived alone” in 2.5% of male and 2.8% of female disclosers who died by firearms.

*Decedents' refusal* (3 male decedents): These decedents refused to give up their firearms, insisting that they needed the firearms for protection.

*Initial removal and then return* (3 male decedents): Families took the decedents' firearms away, but one decedent used an excuse to get his back, and the other's family gave it back to the decedent as he became angry and threatened to hurt the family.

*Inability to find firearms* (3 male decedents): Family was aware that the decedent owned a gun but could not find it.

*Partial removal* (45 male and 4 female decedents): Family members thought that they removed all firearms or ammunition; however, they later found out that the decedents apparently had hidden a firearm or ammunition. Some families were aware that there were more firearms than were removed, but the decedents would not tell them where they kept those hidden. Some families also moved decedents' firearms to a difficult-to-reach place (e.g., basement or attic) because they did not think that decedents, given their disability, would be able to retrieve them. Other families kept firearms in a locked storage/room, but the decedents broke the locks. In a couple of cases, families handed over the decedents' firearms to a law enforcement agent, but the decedents had others. In one case, all handguns, but not rifles, were removed as it was assumed that that the decedent would not use the latter. One decedent used a gun that was kept at his business.

*Purchase of a new firearm close to the injury time* (20 male and 2 female decedents): Family members removed all firearms, but decedents purchased new ones. Some decedents did not previously own a firearm but they bought one close to the injury time without the family's knowledge. Families found purchase receipts that the decedents left behind.

*Means substitution* (8 male decedents): Four male decedents used firearms when families removed knives or pills following their initial suicide attempt using these means. Four other male decedents died by hanging or gas poisoning after their families removed firearms.

## Discussion

In this paper, we focused on suicide decedents age 75+ who had disclosed their suicidal intent (21.4% of suicide decedents age 75+) to examine demographic and clinical factors associated with firearm vs. other method use and to explore firearm safety and access restriction attempts by family members. We found that 82.9% of male disclosers and 27.5% of female disclosers used firearms. Nearly three quarters of all intent disclosers who used firearms disclosed their intent to their family members. Thus, it was important to explore firearm safety and access restriction attempts by family members. This study was the first to do so.

Our findings show that 96% of those who used firearms were non-Hispanic white males, and 74% of those died by firearms, compared to 59% of those who died by other method, had physical health problems as a suicide precipitant. The prominence of physical health problems as a late-life suicide precipitant was expected, as previous studies found terminal illnesses, unremitting pain, and other untreated/worsening health problems were significant contributors to late-life suicide, firearm suicide in particular (9, 42–44). However, our multivariable findings did not show that those with physical health problems had a higher likelihood of using firearms than other methods. This may be due to the fact that we focused on the discloser-decedents age 75+, as physical health problems were more likely to be suicide precipitants in older age groups regardless of suicide method. Our findings of the higher likelihood of firearm use in the South and West compared to the Northeast are consistent with higher firearm suicide rates in these regions that have higher gun ownership rates (45, 46).

This study's key findings are how families did or did not attempt to limit the decedents' access to firearms following decedents' disclosure of their suicidal intent. Although CME/LE narratives included any mention of these attempts or lack thereof for only 11% of firearm suicide decedents, these narratives provided valuable insights and lessons related to means restriction. First, it is important to take older adults' suicidal intent disclosure seriously and take actions to remove potentially lethal means. Second, many older-adult decedents owned multiple guns, which was not surprising as a majority (about two thirds) of gun owners in the US have multiple guns (47). Family members either did not know that or failed to find all of them as older adults who were intent on dying hid at least one firearm. This shows the importance of accounting for all firearms in the household. Third, while family members tried to limit access by moving the firearms to a hard-to-reach place, somehow older adults were able to retrieve them. This indicates the importance of taking firearms away from home. Fourth, means restrictions could not be done for older adults who lived alone or with a disabled spouse, showing the need for involvement of formal support systems. Fifth, rejected assisted suicide requests did not stop some older adults from choosing firearm suicides instead.

Sixth, even when family members succeeded in limiting access to firearms, older adults bought new ones. The ease with which a suicidal older adult could purchase a gun is a fundamental barrier to firearm access restriction. The number of states with Extreme Risk Protection Orders, commonly known as “red flag” laws, has increased since 2018 (to 20 as of June 2023). Red flag laws provide legal authority to temporarily remove firearms and ammunition from a person who demonstrates immediate or imminent risk for gun violence or prohibit the person from purchasing a firearm (48), and were found to have had positive impact in preventing firearm suicides in Connecticut and Indiana that adopted the laws early on (49). However, many states are without the laws and unlikely to adopt it as they are viewed to infringe on the Second Amendment right to bear arms (50). Even in states with red flag laws, those who can file petitions for gun removal are still largely law enforcement officers. Many family members may not be even aware of red flag laws. Recent data also showed that gun ownership increased rapidly during the COVID pandemic in 2020, with 18% of US households purchasing a gun since the start of the pandemic (March 2020–March 2022) in nearly equal parts by people purchasing a gun for the first time and existing gun owners purchasing additional firearms (51, 52). A study of firearm sales showed that new firearm owners were twice more likely than those who did not own firearms to report lifetime, past-year, and past-month suicidal ideation and that half of new owners were women (53).

A systematic review of studies that explored the associations between firearm-related laws and firearm homicides, suicides, and unintentional injuries/deaths show that legislations to restrict access to and regulate firearms saved lives in different countries (54). For example, following the enactment of the 1996 restrictive gun laws and buyback programs, firearm suicide rates in Australia declined significantly, without any evidence of substitution of other lethal methods (55). Laws regulating ownership of a firearm in Sweden and Norway were also associated with decreased firearm suicides (56, 57). In Sweden, a physician is obligated to report a patient to the police if it is suspected that the patient is not fit to possess a firearm because of medical issues (57). In other European countries, firearm availability restrictions were associated with significant downward trends in firearm suicide and homicide rates (58, 59). In the US, a panel of state-level data for the years 1995–2004 showed that gun control measures such as permit and licensing requirements had a negative effect on male suicide rates (60). Another study based on 2010 state data found that states with laws related to permit to purchase a handgun, registration of handguns, and/or license to own a handgun in place exhibited lower overall suicide rates and suicide by firearms rates (61). The results further showed that a smaller proportion of suicides in such states resulted from firearms (61), underscoring the importance of restrictive gun laws for reducing firearm suicides.

Seventh, previous studies found individual-level means substitution to be difficult to assess (62, 63), and NVDRS data do not reveal the full extent of means substitution. CME/LE narratives showed that at least four decedents used other means when families removed firearms, and four others died by firearms when families removed other means. We have no way of knowing how many older adults' lives were saved because their families were successfully able to restrict their access to firearms. However, our findings show that many older adults who disclosed their suicidal intent had a strong intent to die and used all means to die.

This study had limitations. First, although a majority of states participated in the 2017–2019 NVDRS, some states did not provide data on all 3 years and others provided only partial data limited to some counties. Thus, the findings are not representative of all US older-adult suicide decedents. Second, the small proportions of CME/LE narratives on means restrictions may be because many families chose not to report on their lack of attempts or unsuccessful attempts due to shock, grief, and/or guilt following their loved ones' suicide. Underreporting may also be attributable to inconsistency with which data were collected and reported to NVDRS. Thus, the findings should not be generalized beyond the cases that we reviewed in the present study, although they provide invaluable insights. Third, NVDRS data do not contain living arrangement at the time of injury/death, but we suspect that many older decedents (than shown in CME/LE narratives) lived alone and did not have informal support systems. More research is needed on the effect of living arrangement and informal support system availability on means restriction. Fourth, future studies of means restriction need to include a living comparison group to better understand strategies and circumstances of means restriction that helped prevent suicide.

## Conclusion

Given the serious public health crises related to increasing firearm suicides in late life, our findings, despite the above data limitations, have some important clinical and policy implications for suicide prevention. First, since a majority of intent disclosures were made to a partner or family member, there is a need to inform and train informal support systems of older adults who are at risk of suicide on effective means safety/access restriction and strategies to prevent means substitution. For example, primary and secondary interventions can target adult children and other family members through social media campaigns and perhaps information provided via primary care settings of adults who have aging parents. Discussions about means restriction are needed as a universal safety plan when older adults experience deterioration in physical and menta/cognitive health. Significant geographic differences in firearm suicides also underscore the importance of targeting the South and West regions.

Second, there is an urgent need for more comprehensive legislative reforms reducing access to firearms by those at risk of suicide. Although the Second Amendment in the US curtails legislation broadly restricting firearm access, laws that strengthen background checks and permit-to-purchase are needed to limit access to those at risk of self-harm, especially those who disclosed their suicidal intent. The 2022 Bipartisan Safe Communities Act, the first major federal gun safety legislation in decades, allows states to use the funding to manage red flag programs. However, as mentioned, red flag laws are in effect in <40% of the states. As firearms are the most and uniquely prevalent suicide means in the US, more attention needs to be paid to gun control measures as suicide prevention strategies (64). In reality, however, for older adults who are intent on ending their life, an extensive coordination among family members, healthcare providers, law enforcement, and firearm dealers is likely needed to adopt individually tailored approaches.

Third, we need to have more discussion about legalization of physician-assisted suicide nationwide for older adults with terminal illness and pain. Finally, for older adults with physical health problems as a contributor to their suicidal intent, more comfort and palliative care for physical health problems and professional mental health counseling, not just pharmacotherapy, and crisis interventions are needed to maintain open and supportive communication and to alleviate psychosocial risk factors. Along with legislative interventions to reduce access to firearms, easily accessible and affordable, high quality healthcare and psychosocial interventions for older adults who disclosed suicidal thoughts and intent are needed.

## Data availability statement

The data analyzed in this study is subject to the following licenses/restrictions: the authors were granted access to the NVDRS-RAD (Restricted Data Access) based on the NVDRS-RAD review committee's review of our proposal. The authors are not allowed to share the data set with unauthorized people. Requests to access these datasets should be directed to nvdrs-rad@cdc.gov.

## Author contributions

NC: Conceptualization, Data curation, Formal analysis, Funding acquisition, Investigation, Methodology, Project administration, Resources, Software, Supervision, Validation, Visualization, Writing—original draft, Writing—review & editing. CM: Methodology, Supervision, Writing—review & editing. BC: Conceptualization, Investigation, Supervision, Writing—original draft, Writing—review & editing.
