# Supplementary material for: Firearm use risk factors and access restriction among suicide decedents age 75 and older who disclosed their suicidal intent
Source: Front Public Health. 2023 Nov 3;11:1255519. doi: 10.3389/fpubh.2023.1255519 (PMC10654963; doi:10.3389/fpubh.2023.1255519)
Supplement: Supplementary file 1 [file Data_Sheet_1.PDF]

Supplemental Table 1. Characteristics of suicide decedents age 75+ who disclosed suicidal intent within last month by sex

	All those who disclosed			Those who disclosed and used firearms		
	Male	Female	p	Male	Female	p
N (%)	1,476 (85.1)	258 (14.9)		1,223 (94.5)	71 (5.5)	
Suicide method			<0.001			
Firearms	82.9	27.5				
Hanging/suffocation	6.4	16.7				
Poisoning	6.4	46.5				
Other[1]	4.3	9.3				
Disclosed suicidal intent to whom (%)						
Previous or current intimate partner and/or other family member	73.4	71.3	0.494	73.8	74.6	1.000
Friend or neighbor	11.0	10.1	0.745	11.3	15.5	0.256
Healthcare worker	7.2	10.9	0.057	6.1	5.6	1.000
Other	13.8	10.9	0.234	14.0	5.6	0.048
Age group (%)			0.829			0.362
75-84 years	67.8	68.6		67.6	73.2	
85+ years	32.2	31.4		32.4	26.8	
Race/ethnicity (%)			<0.001			0.686
Non-Hispanic white	94.0	87.6		96.0	94.4	
Black/African American	1.7	2.7		1.7	2.8	
Hispanic	2.4	2.3		1.4	1.4	
Asian/Pacific Islander	1.5	7.0		0.4	1.4	
Other	0.5	0.4		0.5	0	
Education (%)			0.165			0.483
=< High school	58.1	51.6		60.3	57.7	
Some college/associate's degree	16.4	20.2		17.1	12.7	
Bachelor's degree or higher	22.8	26.4		20.0	25.4	
Unknown	2.6	1.9		2.6	4.2	
Marital status (%)			<0.001			0.009
Married	48.5	27.1		49.1	28.2	
Widowed	28.5	45.0		28.8	45.1	
Divorced/separated	18.4	22.9		18.2	22.5	
Never married/nonspecified single	3.9	4.3		3.3	2.8	
Missing	0.7	0.8		0.7	1.4	
Military service history (%)	56.2	3.1	<0.001	59.0	5.6	<0.001
Census region (%)			0.009			.118
Northeast	12.5	10.9		10.1	8.5	
Midwest	27.2	18.2		27.7	14.1	
South	26.0	26.7		28.3	35.2	
West	33.8	43.8		33.9	42.3	
Puerto Rico	0.5	0.4		0.1	0	
Injury location (%)			0.547			1.000
At home	87.3	86.0		88.6	88.7	
Not at home	12.7	14.0		11.4	11.3	
History of suicidal thoughts and plans	62.9	65.9	0.401	62.1	60.6	0.803
History of suicide attempt (%)	8.3	22.5	<0.001	5.4	15.5	0.002
Depressed mood at the time of injury	45.9	40.3	0.104	45.4	26.8	0.002
Any diagnosed mental illness[2] (%)	33.4	51.2	<0.001	30.9	40.9	0.080
Depressive disorder/dysthymia (%)	24.9	37.2	<0.001	23.1	32.4	0.084
Bipolar disorder (%)	0.9	5.8	<0.001	0.8	4.2	0.030
Anxiety disorder (%)	5.4	13.2	<0.001	4.7	9.9	0.081
Post-traumatic stress disorder (%)	0.9	0.8	1.000	1.0	0	1.000
Alcohol problem/addiction (%)	5.6	2.7	0.065	5.7	0	0.029
Other substance use problem[2] (%)	1.3	2.7	0.094	1.0	2.8	0.177
Mental health/substance use treatment receipt at the time of injury[3] (%)	17.0	28.7	<0.001	15.3	22.5	0.128
Suicide precipitating factors (%)						

	Physical health problem[4]	72.0	56.6	<0.001	74.2	62.0	0.027
	Suicide/death of spouse/family or traumatic anniversary	14.7	17.8	0.221	14.3	19.7	0.225
	Relationship problem[5]	11.1	12.8	0.456	11.4	12.7	0.702
	financial problem or eviction/loss of housing	6.0	7.4	0.400	5.8	8.5	0.308
	Criminal or civil legal problem	2.2	1.6	0.642	2.2	2.8	0.671
CME/LE narrative search results related to physical health problems							
	Pain	29.6	29.8	0.288	27.5	32.4	0.413
	Cancer	25.2	10.9	<0.001	26.2	9.9	0.001
	Heart disease	17.2	12.4	0.056	17.9	5.6	0.005
	Dementia	12.3	10.5	0.468	11.8	7.0	0.336
	Chronic obstructive pulmonary disease	6.5	6.6	1.000	6.8	4.2	0.621
	Parkinson's	3.3	1.2	0.073	2.9	1.4	0.718
	Worry about becoming a burden	6.0	5.4	0.886	5.9	4.2	0.794
	Refusal of nursing home placement	5.1	3.9	0.440	5.1	1.8	0.575
	Low quality of life	1.6	1.2	0.787	1.4	0	1.000
	Lived alone	2.5	1.9	0.826	2.5	2.8	0.702

Note: P-values are calculated based on omnibus Pearson's χ^2 or Fisher's Exact tests and refer to any differences between sexes,

[1]Jump from a high place, blunt force from moving vehicle/train/other, sharp or blunt object, drowning, smoke/fire/flame/electrocution/hypothermia, other means, or unknown

[2]Including those disorders and syndromes listed in the Diagnostic and Statistical Manual of Mental Disorders, Fifth Edition (DSM-5) with the exception of alcohol and other substance dependence

[3]Inclusive of pharmacotherapy, psychotherapy/counseling, any class (e.g., anger management) attendance, any facility-based care, and alcohol or narcotics anonymous

[4]Including any terminal/other illness, debilitating condition, chronic/acute pain, or other physical/functional issue (perceived, or diagnosed) that were relevant to suicide

[5]Problems with intimate partner and/or other family/relatives, other family stressors, caregiver burden, arguments, or abuse by a caregiver
